# Early retinal and choroidal changes in the macula region associated with axial length in myopic children and adolescents based on swept-source OCTA

**DOI:** 10.3389/fmed.2025.1687058

**Published:** 2026-01-09

**Authors:** Yuan Cao, Qiao Gao, Jun Zhu, Fang Chen

**Affiliations:** 1 Department of Ophthalmology, Northern Jiangsu People’s Hospital Affiliated to Yangzhou University, Yangzhou, China; 2 School of Medicine, Yangzhou University, Yangzhou, China; 3 Department of Ophthalmology, First Affiliated Hospital, Harbin Medical University, Harbin, China

**Keywords:** axial length, myopia, swept-source OCTA, children and adolescents, choroid

## Abstract

**Purpose:**

The study aimed to investigate the association between axial length (AL) and early retinal/choroidal structural and hemodynamic changes in the macula of myopic children and adolescents using swept-source optical coherence tomography angiography (ssOCTA).

**Methods:**

This cross-sectional study included 136 eyes from 136 myopic participants aged 4–17 years. The participants underwent a comprehensive ophthalmic examination, including AL measurement and 6×6 mm macular ssOCTA scans. Hemodynamic parameters (vessel density and choroidal vessel/stromal volumes) and thickness metrics across retinal and choroidal layers and Early Treatment of Diabetic Retinopathy Study (ETDRS) subfields were analyzed. The participants were grouped based on AL (<26 mm vs. ≥26 mm). Multivariate linear regression (MLR; adjusted for age and sex) was used to assess correlations between AL and OCTA parameters.

**Results:**

Increased AL was significantly correlated with reduced deep retinal vessel density (DVD) in the majority of macular regions (except for the perifoveal inferior region), as well as decreased choroidal vessel volume (CVV) and choroidal stromal volume (CSV). Superficial retinal vessel density (SVD) and choriocapillaris flow showed no significant correlation with AL. Retinal and choroidal thickness significantly decreased with longer AL in the majority of regions (except for the foveal region of the retina). Regional analysis revealed that the perifoveal nasal area exhibited the greatest reduction in CVV and CSV, while the perifoveal inferior area showed the least reduction in DVD. Foveal retinal thickness also decreased to a lesser extent compared to other regions with AL elongation.

**Conclusion:**

Axial elongation in young individuals with myopia is associated with specific early microvascular and structural alterations that can be detected using ssOCTA. Deep retinal vascular attenuation and choroidal volumetric loss (CVV, CSV) represent the most prominent hemodynamic changes linked to AL, showing regional variations within the macula. These findings highlight early structural and hemodynamic changes associated with myopic progression in pediatric and juvenile populations.

## Introduction

Myopia is a common yet complex vision disorder with a rising prevalence, particularly among school-aged children in East Asia, posing significant public health challenges (1). Recent studies have pointed out that the global prevalence of myopia has increased over the past decades, rising from 24.32 to 35.81%. It is projected to reach 39.80% by the year 2050 (2). It is widely acknowledged that high myopia permanently alters ocular structures, leading to a range of eye disorders in adulthood (3). Recent research conducted by Jiang et al. further reported that myopic maculopathy could affect as many as 14.05% of highly myopic adolescents, demonstrating the critical need for early prevention and intervention to hinder disease progression (4, 5).

The high incidence of myopia and the occurrence of myopic retinal changes during childhood and adolescence have drawn considerable attention (6), and the structural changes underlying myopia progression have become a hotspot in research (7). With the advent of advanced technologies, including swept-source optical coherence tomography angiography (ssOCTA), it is now possible to visualize and better understand the structural changes underlying myopia progression. Current evidence from ssOCTA scanning indicates that myopia progression is linked to changes in retinal and choroidal structures, such as reduced choroidal thickness and modified choroidal vascular dynamics (8, 9). However, previous studies have primarily focused on general morphological changes (10), with limited exploration of the specific correlations between axial elongation and detailed hemodynamic and thickness metrics across distinct layers and regions of the retina and choroid, particularly in school-aged children and adolescents (8, 11).

This study aimed to address these issues by investigating the relationship between axial length (AL) and variations in macular retinal and choroidal thickness, as well as blood flow, in children and adolescents. It also sought to identify the specific regions and layers most significantly affected by axial growth. By adjusting for potential confounding variables such as age and sex, this research employed multivariate linear regression to analyze the correlation between axial length and retinal and choroidal parameters. Furthermore, it compared the effect of axial length on different retinal and choroidal regions. The findings help enhance our understanding of early structural and hemodynamic changes in the macula region associated with axial elongation, offering valuable insights into the pathophysiology of myopia progression in children and adolescents.

## Participants and methods

### Study participants

**Figure 1:**
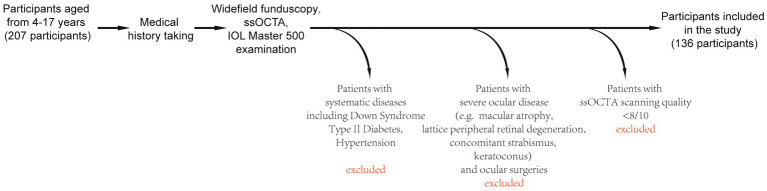
Flowchart illustrating the inclusion and exclusion criteria for study participants.

This cross-sectional, single-center, retrospective study was conducted in Northern Jiangsu People’s Hospital from November 2024 to May 2025. All patients who participated were informed of the purpose of this study before undergoing comprehensive ocular examinations. All patients underwent comprehensive examinations, including the following: (1) Silt lamp examination, (2) scanning laser ophthalmoscopy (SLO), (3) axial length measurement using IOLMaster 500 (Carl Zeiss Meditec AG, Jena, Germany), (4) macular ssOCTA, (5) keratometry analysis, and (6) tropicamide-induced cycloplegic refraction. The exclusion criteria for patients were as follows: (1) Severe fundus diseases, including chorioretinal atrophy associated with high myopia, macular atrophy, lattice peripheral retinal degeneration, primary open-angle glaucoma, and retinal breaks; (2) other ocular diseases, including concomitant strabismus, keratoconus, and keratitis; (3) history of previous ocular surgeries; (4) systematic diseases such as Down syndrome, type 1 diabetes, and hypertension; and (5) ssOCTA scanning quality score of less than 8 out of 10 (Figure 1). All patients were enrolled using their right eye, but when the scan quality of the right eye did not meet the criteria, the patient’s left eye was included for analysis.

### ssOCTA measurements

**Figure 2:**
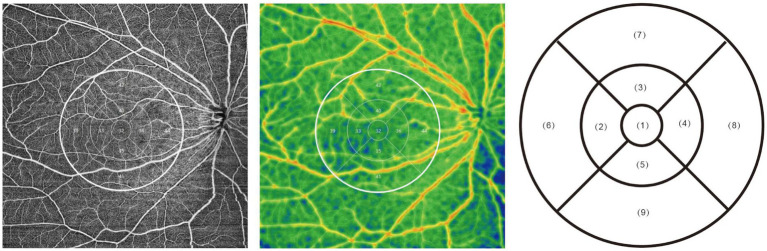
Representative ssOCTA images showing the ETDRS grid and all nine subfields. The OCTA plots on the left and in the middle are from the same participant, showing the raw angiography and quantification of retinal vessel density, respectively, with the 6*6 mm ETDRS region marked by white circles. The 6*6 mm ETDRS grid plot on the right shows the nine grids that were analyzed: (1) foveal, (2) parafoveal temporal, (3) parafoveal superior, (4) parafoveal nasal, (5) parafoveal inferior, (6) perifoveal temporal, (7) perifoveal superior, (8) perifoveal nasal, and (9) perifoveal inferior.

Measurements using ssOCTA were conducted on 136 participants who met the inclusion criteria. Using the cutting-edge 400 kHz full range OCT scanner (BMizar, TowardPi Technology Co Ltd., Beijing, China), we acquired data regarding all layers of the retina and choroid within a 6*6 mm fovea-centered area. Blood flow metrics included superficial retinal vessel density (SVD), deep retinal vessel density (DVD), full vessel density, choriocapillaris vessel density, choroidal medium and large vessel density [from 31 μm below Bruch’s membrane (BM) to the choroidal–scleral interface (CSI)], choroidal vessel volume (CVV, from 31 μm below BM to the CSI), and choroidal stromal volume (CSV, from 31 μm below BM to the CSI). Thickness data included inner retinal thickness [from the inner limiting membrane (ILM) to the inner plexiform layer (IPL)], outer retinal thickness (from the IPL to BM), full retinal thickness (from the ILM to BM), and choroidal thickness (from the BM to the CSI). Based on the traditional Early Treatment of Diabetic Retinopathy Study (ETDRS) grid (12, 13), the area was divided into the fovea area (diameter = 1 mm), the parafoveal ring (diameter 1 ~ 3 mm), and the perifoveal ring (diameter 3 ~ 6 mm). The parafoveal and perifoveal rings were further divided into four quadrants: superior, inferior, temporal, and nasal (Figure 2). All of the above data were automatically calculated using the BMizar 400 kHz full range OCT scanner, and the algorithms for automatic stratification and calculation have been widely accepted in previous research within this field (14, 15).

### Statistical analysis

All data analyses were conducted using R software (version 4.4.2). Data were presented as mean ± SD (standard deviation) that followed a normal distribution and as median (lower quartile, upper quartile) for variables with a non-normal distribution. For normally distributed data, *t*-tests or one-way ANOVA were employed, whereas for non-normally distributed data, Mann–Whitney *U* tests or Kruskal–Wallis *H* tests were applied. The R package *CompareGroups* (version 4.9.1) was utilized for intergroup comparisons. Multivariate linear regression analyses were performed with the R package stats (version 4.4.0). The non-parametric estimation of *b*_AL_ was carried out using the bootstrap method from the R package *boot* (version 1.3). A *p*-value of <0.05 was considered statistically significant.

## Results

### Demographic and clinical statistics of the study participants

**Figure 3:**
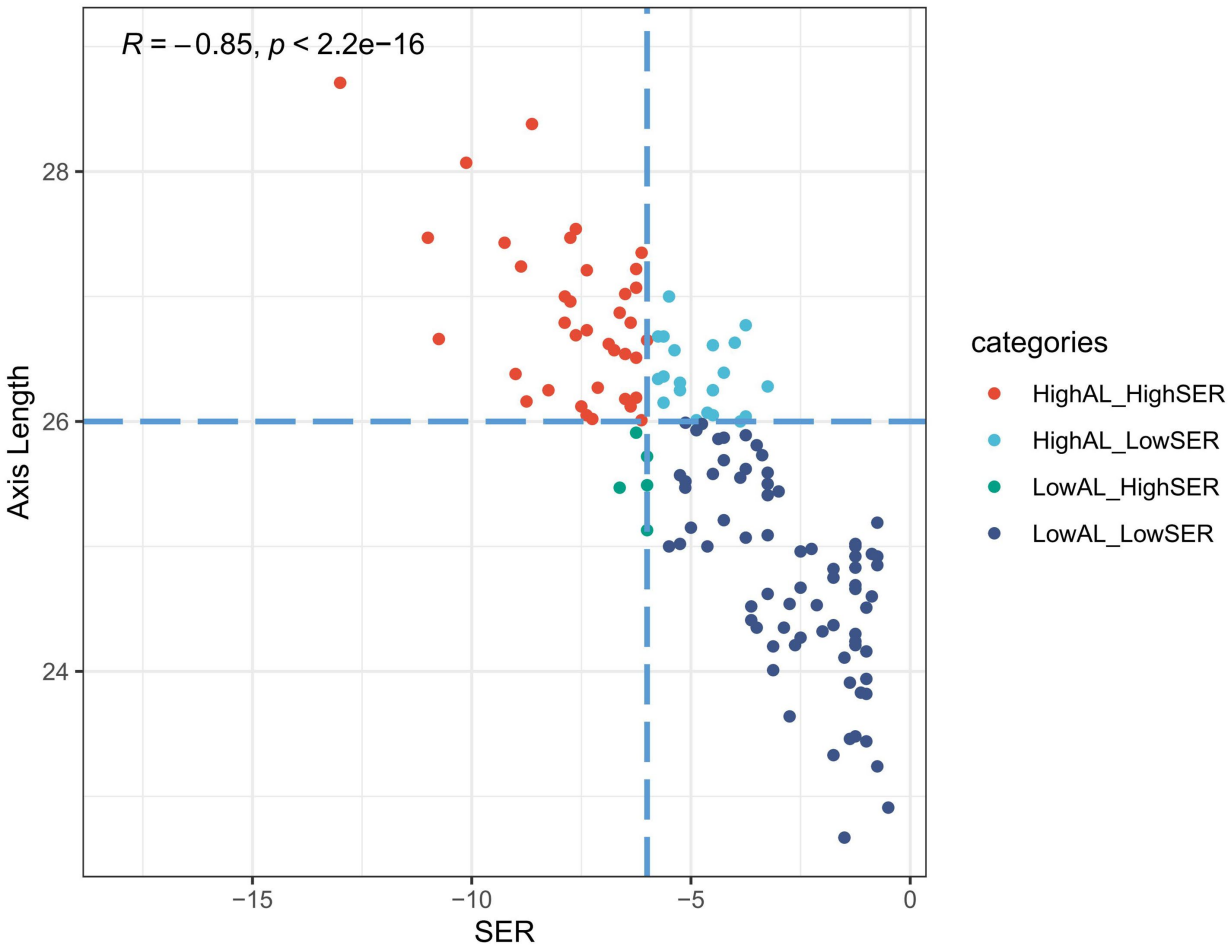
Scatter plot revealing the correlation between AL and SER in the study participants.

Overall, 136 eyes from 136 patients were included in our study (60 male and 76 female individuals). The enrolled study participants were categorized into two groups: one group with an AL ≥ 26 mm and another group with an AL < 26 mm, using 26 mm as the cutoff value. The comparison between these two groups showed significant differences in age and spherical equivalent refraction (SER). The participants in the higher AL group had higher SER and were older (Table 1). Overall, AL showed a significant correlation with SER (Spearman *R* = −0.85, *p* < 0.001) (Figure 3). Although a strong correlation between AL and SER was observed, 20 eyes exhibited relatively high axial length values (with AL ≥ 26 mm), despite not being in the HM group.

**Table 1:** Comparison of demographic data, AL, and SER among low, moderate, and high myopia groups.

Demographic/clincal variables	All patients	AL > =26 mm	AL < 26 mm	*p*-value
Patient sex [n, (%)]				0.281
Male individuals	60 (44.1%)	22 (37.9%)	38 (48.7%)	
Female individuals	76 (55.9%)	36 (62.1%)	40 (51.3%)	
SER (D)	−4.44 (−6.25; −2.44)	−6.38 (−7.62; −5.41)	−2.75 (−4.16; −1.25)	<0.001
AL (mm)	25.6 ± 1.17	26.7 ± 0.59	24.8 ± 0.78	<0.001
Patient age (years)	11.0 (9.00; 12.0)	12.0 (11.0; 14.0)	10.0 (9.00; 11.0)	<0.001

Data with a normal distribution were presented as mean ± SD. Data with a non-normal distribution were presented as median (lower quartile, upper quartile).

### Comparison of ssOCTA hemodynamic data across different AL groups

When comparing retinal and choroidal hemodynamic features between the two groups (AL ≥ 26 mm and AL < 26 mm), three specific parameters showed significant differences, namely deep retinal vessel density (DVD), the luminal volume of the choroidal medium- and large-vessel layers (choroidal vessel volume, CVV), and the stromal volume of the same choroidal medium- and large-vessel layers (choroidal stromal volume, CSV). In contrast, there were no significant differences in superficial retinal vessel density (SVD), choriocapillaris vessel density, or the density of choroidal medium- and large-vessel layers (Table 2).

**Table 2:** Participants were divided into two groups based on an axial length of 26 mm.

ETDRS region	All patients	AL > =26 mm	AL < 26 mm	*p*-value
Superficial retina				
Fovea	34.3 ± 3.29	34.2 ± 3.51	34.4 ± 3.14	0.651
Parafoveal temporal	37.5 (36.0; 39.0)	37.0 (36.0; 39.0)	38.0 (37.0; 39.0)	0.211
Parafoveal superior	40.0 (38.0; 42.0)	40.0 (38.0; 41.0)	40.0 (38.0; 42.0)	0.321
Parafoveal nasal	38.0 (37.0; 40.0)	38.0 (36.0; 39.0)	39.0 (37.0; 40.8)	0.026
Parafoveal inferior	40.0 (37.8; 41.0)	40.0 (38.0; 41.8)	39.0 (37.0; 41.0)	0.288
Perifoveal temporal	43.0 (42.0; 44.2)	43.0 (42.0; 44.0)	43.0 (42.0; 45.0)	0.235
Perifoveal superior	44.0 (43.0; 45.0)	44.0 (42.2; 45.0)	44.0 (43.0; 45.8)	0.162
Perifoveal nasal	43.0 (41.0; 44.0)	43.0 (41.0; 44.0)	43.0 (41.0; 44.0)	0.964
Perifoveal inferior	44.0 (43.0; 45.0)	44.0 (43.0; 45.0)	44.0 (43.0; 46.0)	0.251
Deep retina				
Fovea	37.0 (33.0; 39.0)	36.0 (32.0; 39.0)	38.0 (35.0; 39.0)	0.048
Parafoveal temporal	45.0 (43.0; 46.0)	44.0 (42.0; 46.0)	46.0 (44.0; 47.0)	<0.001
Parafoveal superior	45.0 (43.0; 46.0)	44.0 (42.2; 45.0)	46.0 (44.0; 46.0)	<0.001
Parafoveal nasal	45.0 (43.0; 46.0)	44.0 (42.0; 45.0)	45.0 (44.0; 46.0)	<0.001
Parafoveal inferior	45.0 (43.0; 46.0)	44.5 (42.0; 46.0)	45.5 (43.0; 47.0)	0.003
Perifoveal temporal	46.0 (44.0; 47.0)	45.0 (44.0; 46.0)	46.0 (45.0; 47.0)	0.003
Perifoveal superior	46.0 (45.0; 47.0)	46.0 (44.0; 46.8)	46.0 (45.0; 47.0)	0.011
Perifoveal nasal	45.0 (44.0; 46.0)	45.0 (43.0; 46.0)	46.0 (44.0; 46.8)	<0.001
Perifoveal inferior	46.0 (44.0; 47.0)	45.5 (44.0; 47.0)	46.0 (45.0; 47.0)	0.170
Choriocapillaris				
Fovea	46.0 (44.0; 49.0)	47.0 (44.0; 49.8)	45.0 (44.0; 48.0)	0.264
Parafoveal temporal	46.0 (45.0; 47.0)	46.5 (45.2; 47.0)	46.0 (45.0; 47.0)	0.555
Parafoveal superior	46.0 (45.0; 47.0)	46.0 (45.0; 47.0)	46.0 (45.0; 47.0)	0.740
Parafoveal nasal	46.0 (45.0; 47.0)	46.0 (45.0; 47.0)	46.0 (45.0; 47.0)	0.077
Parafoveal inferior	46.0 (45.0; 48.0)	46.0 (46.0; 48.0)	46.0 (45.0; 48.0)	0.314
Perifoveal temporal	49.0 (48.0; 50.0)	49.0 (48.0; 50.0)	49.0 (48.0; 50.0)	0.271
Perifoveal superior	48.0 (48.0; 49.0)	49.0 (48.0; 49.0)	48.0 (47.2; 49.0)	0.196
Perifoveal nasal	47.5 (47.0; 48.0)	48.0 (47.0; 49.0)	47.0 (47.0; 48.0)	0.003
Perifoveal inferior	49.0 (47.0; 50.0)	48.5 (47.0; 50.0)	49.0 (47.0; 50.0)	0.989
Choroidal medium- and large-vessel density				
Fovea	50.0 (47.0; 56.0)	52.0 (49.0; 57.0)	49.0 (47.0; 54.8)	0.019
Parafoveal temporal	51.0 (48.0; 57.0)	53.0 (49.0; 57.0)	50.0 (48.0; 56.0)	0.179
Parafoveal superior	50.0 (48.0; 57.0)	54.0 (48.0; 57.8)	50.0 (47.0; 57.0)	0.152
Parafoveal nasal	50.0 (47.0; 56.0)	53.0 (48.0; 57.0)	49.0 (47.0; 55.0)	0.020
Parafoveal inferior	50.0 (47.0; 57.2)	53.5 (47.2; 58.0)	50.0 (47.0; 57.0)	0.172
Perifoveal temporal	51.0 (49.0; 58.0)	51.0 (49.0; 57.8)	52.0 (49.0; 58.0)	0.472
Perifoveal superior	50.0 (48.0; 57.0)	54.5 (48.0; 58.0)	50.0 (48.0; 57.0)	0.283
Perifoveal nasal	51.0 (49.0; 57.0)	52.0 (49.0; 56.0)	50.0 (48.0; 57.0)	0.321
Perifoveal inferior	51.0 (49.0; 58.0)	55.0 (49.0; 58.0)	51.0 (49.0; 57.0)	0.442
Choroidal medium- and large-vessel volume (CVV)				
Fovea	68.0 (47.0; 88.0)	56.5 (41.5; 83.0)	75.5 (61.0; 92.8)	0.003
Parafoveal temporal	114 (86.0; 148)	99.5 (74.5; 136)	126 (95.5; 155)	0.005
Parafoveal superior	130 (102; 163)	119 (89.0; 150)	138 (112; 168)	0.018
Parafoveal nasal	128 (95.5; 165)	114 (79.2; 157)	146 (109; 170)	0.002
Parafoveal inferior	134 (96.8; 175)	106 (79.5; 160)	146 (114; 182)	0.001
Perifoveal temporal	276 (195; 376)	262 (171; 348)	286 (239; 409)	0.045
Perifoveal superior	428 (348; 524)	393 (326; 492)	452 (376; 552)	0.040
Perifoveal nasal	417 ± 160	362 ± 172	458 ± 139	0.001
Perifoveal inferior	397 (319; 517)	354 (272; 463)	416 (358; 534)	0.004
Choroidal medium- and large-vessel stromal volume (CSV)				
Fovea	78.0 (63.5; 95.0)	70.0 (54.0; 80.5)	82.0 (73.0; 104)	<0.001
Parafoveal temporal	133 (110; 166)	120 (94.0; 150)	142 (120; 184)	0.001
Parafoveal superior	164 (138; 186)	152 (122; 169)	172 (154; 207)	<0.001
Parafoveal nasal	174 ± 53.9	152 ± 50.1	190 ± 51.1	<0.001
Parafoveal inferior	157 (130; 186)	134 (117; 169)	164 (142; 205)	<0.001
Perifoveal temporal	338 (255; 485)	312 (208; 501)	348 (286; 480)	0.093
Perifoveal superior	573 (481; 671)	538 (428; 624)	596 (540; 720)	0.010
Perifoveal nasal	605 ± 195	539 ± 205	654 ± 173	0.001
Perifoveal inferior	519 (440; 612)	476 (395; 572)	536 (490; 649)	0.001

Comparisons between the two groups for different hemodynamic parameters were conducted.

### Comparison of ssOCTA thickness data across different AL groups

A comparison of thickness between the two groups (AL ≥ 26 mm and AL < 26 mm) revealed only a slight decrease in inner retina thickness in some regions (parafoveal nasal, parafoveal inferior, perifoveal temporal, and perifoveal superior) in the AL ≥ 26 mm group. However, the thickness of almost all regions in the outer retina and choroid was significantly lower in the AL ≥ 26 mm group, except for the fovea region in the outer retina (Table 3).

**Table 3:** Participants were divided into two groups based on an axial length of 26 mm.

ETDRS region	All patients	AL > =26 mm	AL < 26 mm	*p*-value
Inner retina thickness				
Fovea	62.5 ± 9.88	63.2 ± 10.0	62.0 ± 9.81	0.471
Parafoveal temporal	121 ± 6.14	120 ± 5.79	122 ± 6.31	0.079
Parafoveal superior	132 (127; 134)	131 (125; 134)	132 (128; 136)	0.222
Parafoveal nasal	130 ± 7.07	128 ± 6.61	131 ± 7.24	0.034
Parafoveal inferior	131 ± 6.54	130 ± 6.32	132 ± 6.54	0.027
Perifoveal temporal	95.9 ± 5.39	93.9 ± 4.89	97.4 ± 5.27	<0.001
Perifoveal superior	112 ± 6.95	111 ± 7.07	113 ± 6.79	0.114
Perifoveal nasal	132 (126; 136)	132 (126; 137)	132 (127; 136)	0.845
Perifoveal inferior	110 ± 7.30	109 ± 7.44	110 ± 7.17	0.231
Outer retina thickness				
Fovea	231 (223; 237)	231 (224; 236)	230 (222; 238)	0.822
Parafoveal temporal	231 (222; 237)	227 (220; 233)	232 (227; 238)	0.004
Parafoveal superior	232 (225; 238)	230 (224; 237)	234 (227; 240)	0.018
Parafoveal nasal	234 (226; 242)	230 (226; 239)	236 (227; 244)	0.021
Parafoveal inferior	226 (218; 231)	223 (217; 227)	229 (219; 234)	0.006
Perifoveal temporal	207 (201; 214)	205 (199; 212)	210 (204; 215)	0.012
Perifoveal superior	207 ± 9.66	204 ± 8.23	209 ± 10.2	0.003
Perifoveal nasal	209 ± 10.1	207 ± 8.15	210 ± 11.1	0.039
Perifoveal inferior	194 (187; 199)	192 (186; 196)	195 (190; 201)	0.010
Choroidal thickness				
Fovea	212 (174; 252)	180 (150; 236)	223 (190; 267)	<0.001
Parafoveal temporal	234 ± 59.7	214 ± 58.0	250 ± 56.4	<0.001
Parafoveal superior	217 (186; 254)	202 (162; 229)	225 (200; 271)	0.002
Parafoveal nasal	179 (146; 214)	162 (132; 194)	190 (166; 230)	<0.001
Parafoveal inferior	208 (177; 257)	182 (154; 238)	228 (198; 270)	<0.001
Perifoveal temporal	240 ± 53.1	223 ± 53.1	253 ± 49.7	0.001
Perifoveal superior	218 (190; 256)	205 (172; 242)	222 (207; 269)	0.015
Perifoveal nasal	136 (112; 163)	122 (96.5; 145)	144 (125; 180)	<0.001
Perifoveal inferior	202 (174; 241)	188 (158; 228)	213 (190; 263)	0.001

Comparisons between the two groups for the thickness parameters of the retina and choroid were conducted.

### Multivariate linear regression of retinal and choroidal vascular metrics

To account for potential confounding factors such as age and sex, a multivariate linear regression (MLR) model was utilized (Table 4). The MLR formula is as follows:

$$\text{Vessel metrics}=b_{\mathrm{AL}}\times \mathrm{AL}+b_{\mathrm{Age}}\times \mathrm{Age}+b_{\text{Gender}}\times \text{Gender}+b_{0}$$

**Table 4:** Multivariate regression analysis of the influence of AL (all adjusted for age and sex) on different hemodynamic parameters.

ETDRS region	Partial regression coefficient (*b_AL_*)	*p*-value	95% confidence interval
Superficial retina			
Fovea	−0.124	0.677	(−0.711, 0.463)
Parafoveal temporal	−0.318	0.200	(−0.808, 0.171)
Parafoveal superior	−0.166	0.484	(−0.635, 0.302)
Parafoveal nasal	−0.364	0.131	(−0.838, 0.109)
Parafoveal inferior	0.344	0.191	(−0.173, 0.862)
Perifoveal temporal	−0.09	0.676	(−0.514, 0.334)
Perifoveal superior	0.012	0.944	(−0.328, 0.352)
Perifoveal nasal	−0.067	0.665	(−0.373, 0.239)
Perifoveal inferior	−0.063	0.770	(−0.485, 0.36)
Deep retina			
Fovea	−1.046	0.011	(−1.845, −0.247)
Parafoveal temporal	−0.781	0.001	(−1.233, −0.329)
Parafoveal superior	−0.826	0.000	(−1.241, −0.411)
Parafoveal nasal	−0.771	0.000	(−1.174, −0.368)
Parafoveal inferior	−0.657	0.004	(−1.095, −0.22)
Perifoveal temporal	−0.376	0.023	(−0.7, −0.053)
Perifoveal superior	−0.475	0.003	(−0.782, −0.167)
Perifoveal nasal	−0.542	0.000	(−0.836, −0.248)
Perifoveal inferior	−0.096	0.559	(−0.419, 0.228)
Choriocapillaris			
Fovea	0.608	0.069	(−0.049, 1.265)
Parafoveal temporal	0.022	0.900	(−0.321, 0.364)
Parafoveal superior	0.006	0.967	(−0.259, 0.27)
Parafoveal nasal	0.084	0.592	(−0.225, 0.393)
Parafoveal inferior	0.257	0.160	(−0.103, 0.617)
Perifoveal temporal	0.35	0.032	(0.03, 0.669)
Perifoveal superior	0.188	0.143	(−0.065, 0.441)
Perifoveal nasal	0.196	0.111	(−0.046, 0.437)
Perifoveal inferior	0.094	0.545	(−0.212, 0.4)
Choroidal medium- and large-vessel density			
Fovea	1.038	0.036	(0.07, 2.005)
Parafoveal temporal	0.455	0.347	(−0.499, 1.409)
Parafoveal superior	0.928	0.051	(−0.005, 1.861)
Parafoveal nasal	1.029	0.033	(0.082, 1.976)
Parafoveal inferior	0.738	0.152	(−0.276, 1.751)
Perifoveal temporal	−0.622	0.341	(−1.907, 0.664)
Perifoveal superior	0.602	0.177	(−0.276, 1.48)
Perifoveal nasal	0.437	0.287	(−0.372, 1.246)
Perifoveal inferior	0.551	0.228	(−0.349, 1.451)
Choroidal medium- and large-vessel volume (CVV)			
Fovea	−7.708	0.003	(−12.738, −2.678)
Parafoveal temporal	−10.816	0.015	(−19.464, −2.167)
Parafoveal superior	−10.442	0.026	(−19.644, −1.24)
Parafoveal nasal	−16.028	0.001	(−25.071, −6.986)
Parafoveal inferior	−17.267	0.001	(−26.91, −7.624)
Perifoveal temporal	−31.347	0.027	(−59.001, −3.693)
Perifoveal superior	−24.827	0.056	(−50.348, 0.695)
Perifoveal nasal	−51.964	0.000	(−80.19, −23.738)
Perifoveal inferior	−38.002	0.008	(−65.99, −10.014)
Choroidal medium- and large-vessel stromal volume (CSV)			
Fovea	−9.938	0.000	(−14.13, −5.747)
Parafoveal temporal	−18.497	0.000	(−27.804, −9.189)
Parafoveal superior	−17.973	0.000	(−26.001, −9.944)
Parafoveal nasal	−18.897	0.000	(−28.069, −9.724)
Parafoveal inferior	−17.487	0.000	(−25.979, −8.994)
Perifoveal temporal	−41.709	0.024	(−77.834, −5.585)
Perifoveal superior	−44.675	0.002	(−72.532, −16.818)
Perifoveal nasal	−60.009	0.001	(−94.584, −25.434)
Perifoveal inferior	−50.182	0.000	(−77.87, −22.495)

The 95% confidence interval (CI) for the partial regression coefficient of AL was also estimated.

In this model, superficial retinal vessel density (SVD) exhibited no significant correlation, while deep retinal vessel density (DVD) demonstrated a significant negative correlation with axial length (AL) across all ETDRS grid areas, except for the perifoveal inferior region. Consistent with the comparison results presented in Table 2, no significant correlation was observed between AL and choriocapillaris vessel density (except in the perifoveal temporal region) or between AL and choroidal medium- and large-vessel density (except in the foveal region). Notably, even after adjusting for potential confounding factors such as age and sex, AL showed a strong negative correlation with choroidal stromal volume (CSV) and choroidal vessel volume (CVV), which contrasted with the findings regarding choroidal vessel density in the MLR analysis.

### Multivariate linear regression of retinal and choroidal thickness metrics

A multivariate linear regression (MLR) model was used to adjust for the influence of cofounding factors such as age and sex (Table 5). The MLR formula is as follows:

$$\text{Thickness}=b_{\mathrm{AL}}\times \mathrm{AL}+b_{\mathrm{Age}}\times \mathrm{Age}+b_{\text{Gender}}\times \text{Gender}+b_{0}$$

**Table 5:** Multivariate regression analysis of the influence of AL (all adjusted for age and sex) on different thickness parameters.

ETDRS region	Partial regression coefficient (*b_AL_*)	*p*-value	95% confidence interval
Inner retinal thickness			
Fovea	0.108	0.900	(−1.591, 1.807)
Parafoveal temporal	−1.341	0.014	(−2.411, −0.271)
Parafoveal superior	−1.869	0.003	(−3.076, −0.663)
Parafoveal nasal	−1.999	0.001	(−3.201, −0.797)
Parafoveal inferior	−1.844	0.002	(−2.992, −0.695)
Perifoveal temporal	−1.915	0.000	(−2.833, −0.998)
Perifoveal superior	−1.446	0.023	(−2.688, −0.204)
Perifoveal nasal	−0.857	0.237	(−2.284, 0.57)
Perifoveal inferior	−1.316	0.052	(−2.646, 0.013)
Outer retina thickness			
Fovea	0.141	0.908	(−2.254, 2.535)
Parafoveal temporal	−1.957	0.075	(−4.112, 0.199)
Parafoveal superior	−1.673	0.082	(−3.563, 0.216)
Parafoveal nasal	−2.301	0.025	(−4.309, −0.293)
Parafoveal inferior	−1.702	0.099	(−3.726, 0.322)
Perifoveal temporal	−2.060	0.029	(−3.907, −0.213)
Perifoveal superior	−2.345	0.006	(−4.003, −0.686)
Perifoveal nasal	−2.518	0.004	(−4.222, −0.815)
Perifoveal inferior	−2.554	0.005	(−4.337, −0.771)
Choroidal thickness			
Fovea	−21.871	0.000	(−32.683, −11.059)
Parafoveal temporal	−20.122	0.000	(−30.341, −9.904)
Parafoveal superior	−17.953	0.001	(−28.254, −7.652)
Parafoveal nasal	−20.474	0.000	(−30.976, −9.972)
Parafoveal inferior	−21.995	0.000	(−32.918, −11.072)
Perifoveal temporal	−17.040	0.000	(−26.22, −7.86)
Perifoveal superior	−13.115	0.009	(−22.836, −3.394)
Perifoveal nasal	−17.731	0.000	(−26.991, −8.471)
Perifoveal inferior	−16.611	0.002	(−26.797, −6.426)

The 95% confidence interval (CI) for the partial regression coefficient of AL was also estimated.

The negative correlation observed between inner and outer retinal thickness and AL reached statistical significance only in some of the ETDRS sections. Not surprisingly, total retinal and choroidal thickness was negatively correlated with AL after adjusting for age and sex, except for retinal thickness in the fovea, which showed no significant correlation.

### Comparison of AL’s influence across different ETDRS areas of retinal and choroidal metrics

**Figure 4:**
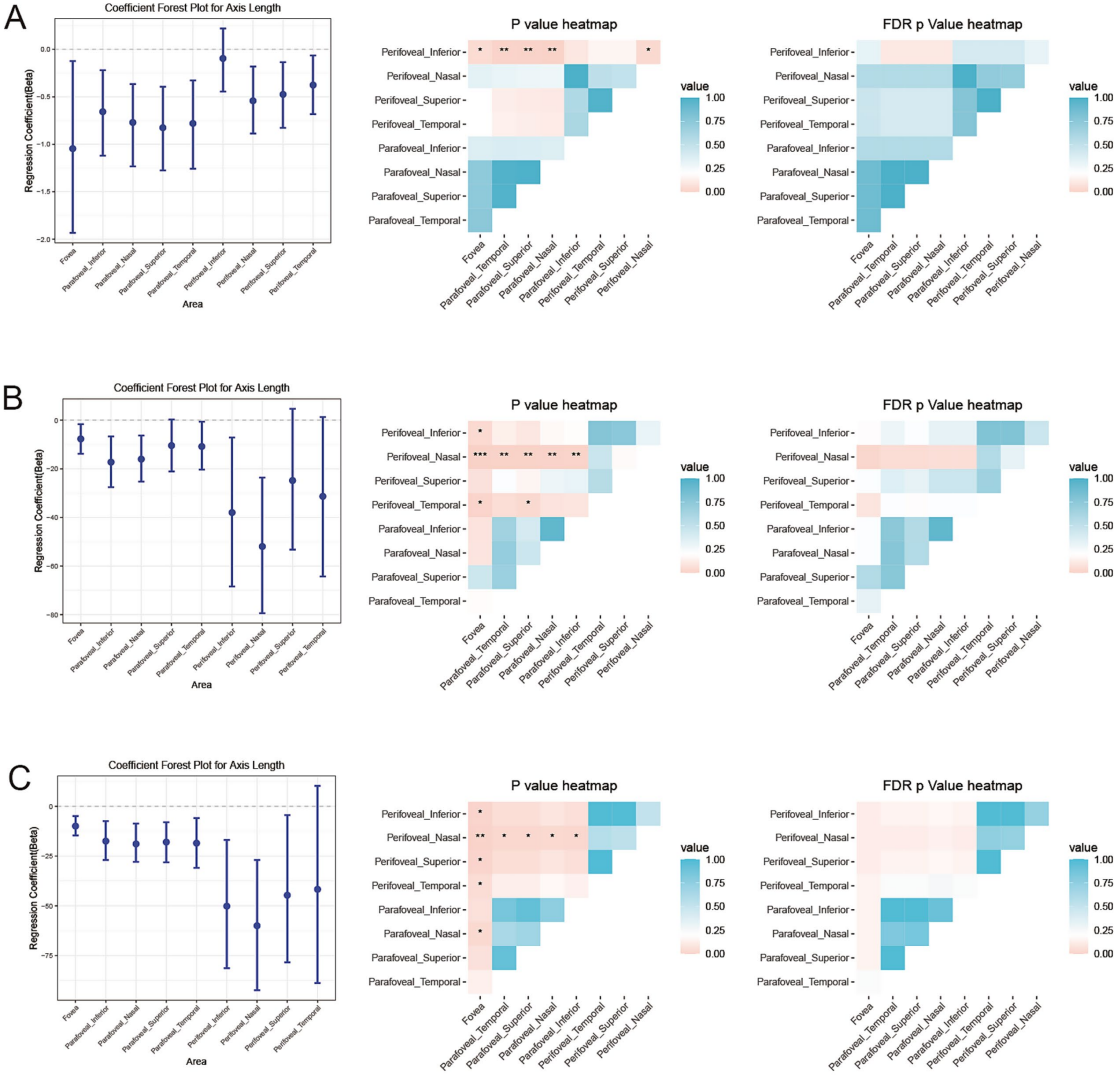
Estimation and comparison of AL’s influence across different ETDRS areas of retinal and choroidal blood flow metrics. Forest plot (showing the estimated value and 95%CI using the bootstrap method) and *p*-value heatmap comparing the partial regression coefficient for AL with **(A)** deep retinal vessel density (DVD), **(B)** choroidal vessel volume (CVV), and **(C)** choroidal stromal volume (CSV) across the nine subfields. *p < 0.05, **p < 0.01, ***p < 0.001.

**Figure 5:**
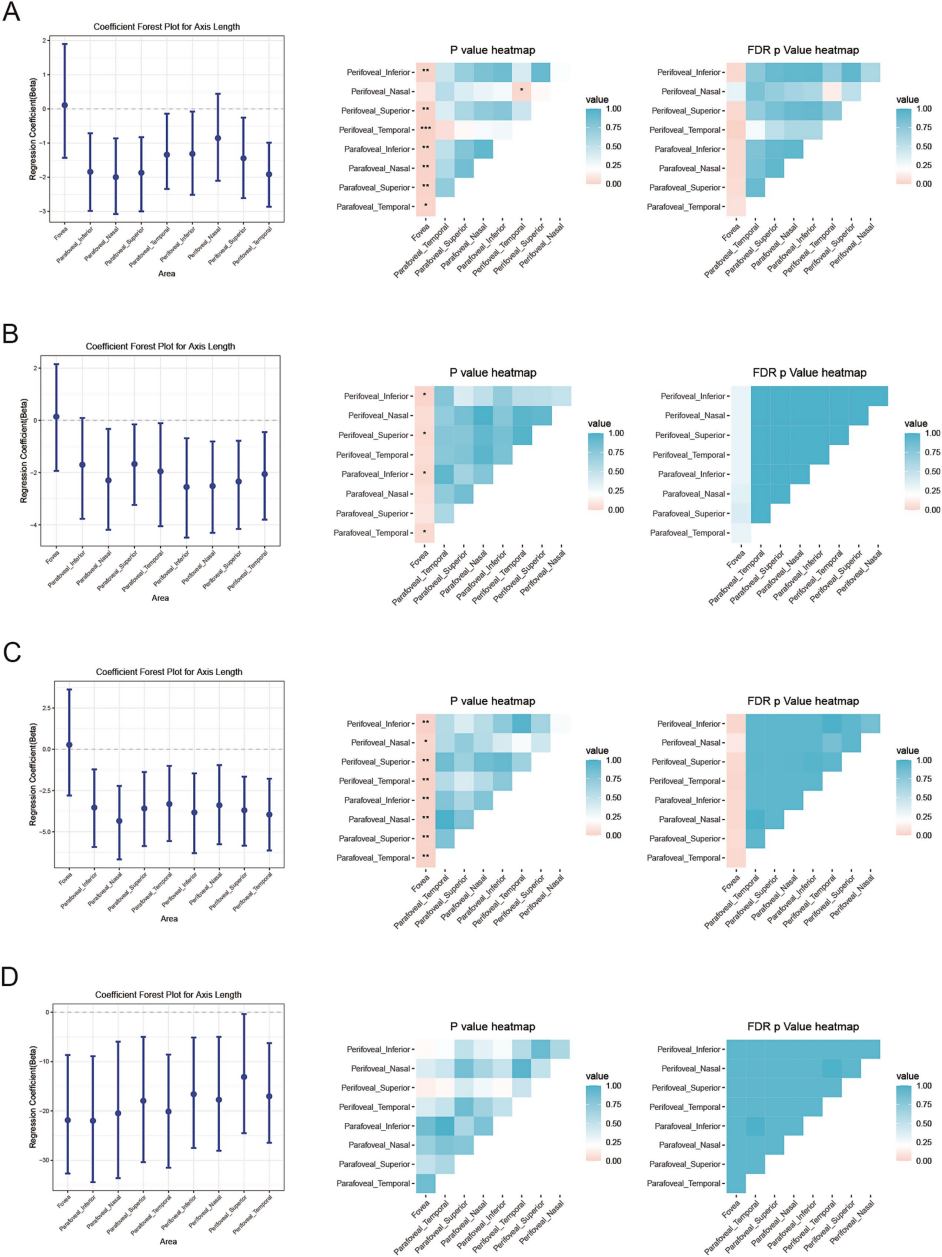
Estimation and comparison of AL’s influence across different ETDRS areas of retinal and choroidal thickness metrics. Forest plot (showing the estimated value and 95%CI using the bootstrap method) and *p*-value heatmap comparing the partial regression coefficient for AL with **(A)** inner retinal thickness, **(B)** outer retinal thickness, **(C)** total retinal thickness, and **(D)** choroidal thickness across the nine subfields. *p < 0.05, **p < 0.01, ***p < 0.001.

To compare the influence of AL across different ETDRS areas, the 95% confidence interval (CI) of the partial regression coefficient of AL (*b*_AL_) was estimated using the bootstrap method, and comparisons between different areas were conducted using likelihood ratio tests with FDR adjustments. DVD, CSV, and CVV were selected for analysis because they showed significant associations with AL. The results were presented using a forest plot and a heatmap. We found that, with increasing AL, the perifoveal inferior area showed a significantly smaller decrease in DVD compared to all other regions (Figure 4A). However, the perifoveal nasal area showed substantially greater decreases in CSV and CVV than most of the other regions (fovea, parafoveal temporal, parafoveal nasal, and parafoveal inferior) (Figures 4B,C). A significantly smaller decrease in foveal retinal thickness compared to other regions with increasing AL was also found (Figure 5).

## Discussion

Research on myopic fundus changes has spanned several decades. In 1970, Curtin et al. first identified five fundus changes related to axial elongation: Chorioretinal atrophy, central pigment spots (Fuchs spots), lacquer cracks, posterior scleral staphyloma, and optic disc abnormalities (16). In 2015, the International META-analysis for Pathologic Myopia Study Group proposed a simplified classification system, categorizing pathological myopia-related macular degeneration into five distinct grades: C0, no myopic retinal degeneration; C1, tigroid fundus; C2, diffuse chorioretinal atrophy; C3, patchy chorioretinal atrophy; and C4, macular atrophy (17). This classification fully revealed the progression of myopic macular degeneration based on fundus photography. Nevertheless, distinct retinal and choroidal changes in the early stages of myopic macular alterations still need further investigation, and ssOCTA has become a valuable tool.

Many studies on retinal and choroidal vascular changes in myopia, based on ssOCTA results have been published, most of which involved participants in adulthood (18–20). Although some exciting discoveries have been made, certain aspects need to be considered when investigating myopic changes in adolescence and childhood. It is widely acknowledged that AL is positively correlated with age in children (21), and sex may potentially influence microvascular metrics (22, 23). Therefore, cofounding factors such as age and sex should be controlled to achieve a convincing result.

In our study, after controlling for the participants’ sex and age using an MLR model, ssOCTA analysis revealed that axial elongation had a more pronounced effect on DVD and choroidal vessel/stromal volume compared to other hemodynamic metrics. This suggests that the microvascular network in the deeper layers of the retina may be more susceptible to the pathophysiological changes associated with axial myopia, which may be attributed to the extension of the retina following AL elongation (24). These findings echo the results obtained by Mu et al., who found that SVD was not associated with axial elongation after adjusting for age and sex, whereas DVD was inversely correlated with axial length (25).

We also found that CSV and CVV were negatively related to AL after controlling for age and sex in the participants aged 4–17 years across all ETDRS sections, and choriocapillaris blood flow remained unaffected by AL elongation. This finding is similar to that of Geng et al., who found a negative relationship between CSV and CVV in myopic individuals aged 4–14 years (26). Moreover, the results support the hypothesis proposed by Jonas et al., who stated that the decrease in choriocapillaris blood flow in previous case–control studies may be due to the inclusion of patients with avascular chorioretinal patchy atrophy (27). Our study further revealed that the perifoveal inferior region showed a stronger decrease in CSV and CVV with increasing AL, highlighting possible regional disparities in choroidal structural changes.

A previous longitudinal study found that distinct retinal layers, such as the ganglion cell layer (GCL), the inner plexiform layer (IPL), and total retinal thickness, showed no significant changes in thickness in myopic adolescents after a 2-year follow-up (28). Nonetheless, we noted that total retinal thickness was negatively correlated with AL, except in the foveal region. This finding is similar to a previous cross-sectional study in young adults (29), suggesting that the possible thinning of the retina in the macular region may begin earlier than expected.

The results also emphasize the value of using advanced imaging techniques such as ssOCTA to elucidate the specific effects of axial elongation on different components of the ocular microvasculature (30). By identifying the areas and layers most affected by axial growth, this study provides insights into potential targets for early intervention strategies aimed at slowing myopia progression in children and adolescents.

This study has a few limitations worth noting. First, due to its cross-sectional design, it is difficult to establish a definitive cause-and-effect relationship. Longitudinal studies are needed to confirm the observed associations and better understand the dynamic changes over time. Furthermore, the hospital-based nature of the study, although adequate for detecting significant differences, may limit the generalizability of the findings to broader populations. Future studies with larger and more diverse cohorts are needed. Finally, the use of a single imaging modality (ssOCTA) may not capture the full spectrum of vascular changes associated with myopia. Combining ssOCTA with other imaging techniques could provide a more comprehensive understanding of structural and hemodynamic alterations.

In conclusion, this study provides a detailed analysis of the relationship between axial length and retinal and choroidal hemodynamic metrics in children and adolescents. The findings indicate that axial elongation is associated with significant changes in specific hemodynamic parameters, particularly deep retinal vessel density and choroidal vessel/stromal volume. These results enhance our understanding of early structural and hemodynamic changes in the macula region associated with axial elongation and offer valuable insights into the pathophysiology of myopia progression in children and adolescents. Future research should focus on longitudinal studies and larger cohorts to further validate these findings and explore their clinical implications.

## Data Availability

The raw data supporting the conclusions of this article will be made available by the authors, without undue reservation.

## References

[1] Morgan IG French AN Ashby RS Guo X Ding X He M . The epidemics of myopia: aetiology and prevention. Prog Retin Eye Res. (2018) 62:134–49. doi: 10.1016/j.preteyeres.2017.09.004, 28951126

[2] Liang J Pu Y Chen J Liu M Ouyang B Jin Z . Global prevalence, trend and projection of myopia in children and adolescents from 1990 to 2050: a comprehensive systematic review and meta-analysis. Br J Ophthalmol. (2025) 109:362–71. doi: 10.1136/bjo-2024-325427, 39317432

[3] Williams K Hammond C. High myopia and its risks. Community Eye Health. (2019) 32:5–6. 31409941 PMC6688422

[4] Ohno-Matsui K. Insights into childhood myopic maculopathy. JAMA Ophthalmol. (2024) 142:186. doi: 10.1001/jamaophthalmol.2023.6490, 38270964

[5] Jiang F Wang D Xiao O Guo X Yin Q Luo L . Four-year progression of myopic maculopathy in children and adolescents with high myopia. JAMA Ophthalmol. (2024) 142:180. doi: 10.1001/jamaophthalmol.2023.6319, 38270935 PMC10811590

[6] Zhang W Yang F Chen S Shi T. Peripheral and posterior pole retinal changes in highly myopic Chinese children and adolescents: retinal changes in Chinese children and adolescents. BMC Ophthalmol. (2024) 24:65. doi: 10.1186/s12886-024-03328-6, 38350965 PMC10865601

[7] Kong K Liu X Fang Z Jiang J Jiang J Wang D . Axial elongation in nonpathologic high myopia: ocular structural changes and glaucoma diagnostic challenges. Asia Pac J Ophthalmol (Phila). (2024) 13:100123. doi: 10.1016/j.apjo.2024.100123, 39674402

[8] Gu J Xu Y Xiao D Wang Y Lei W Chen Z . Peripheral superficial retina vascular density and area of radial peripapillary capillaries changes in myopic individuals: a wide-field OCT angiography study. Transl Vis Sci Technol. (2024) 13:21. doi: 10.1167/tvst.13.9.21, 39292467 PMC11412621

[9] Jiang Z Hou A Zhang T Lai Y Huang L Ding X. Pattern of choroidal thickness in early-onset high myopia. Front Med. (2023) 10:1156259. doi: 10.3389/fmed.2023.1156259, 37538314 PMC10394095

[10] Zhang JM Wu JF Chen JH Wang L Lu TL Sun W . Macular choroidal thickness in children: the Shandong children eye study. Invest Ophthalmol Vis Sci. (2015) 56:7646–52. doi: 10.1167/iovs.15-17137, 26624496

[11] Jonas JB Spaide RF Ostrin LA Logan NS Flitcroft I Panda-Jonas S. IMI-nonpathological human ocular tissue changes with axial myopia. Invest Ophthalmol Vis Sci. (2023) 64:5. doi: 10.1167/iovs.64.6.5, 37126358 PMC10153585

[12] Liu R Xuan M Wang D-C Xiao O Guo X-X Zhang J . Using choroidal thickness to detect myopic macular degeneration. Int J Ophthalmol. (2024) 17:317–23. doi: 10.18240/ijo.2024.02.14 38371267 PMC10827620

[13] Burguera-Giménez N Díez-Ajenjo MA Burguera N Briceno-Lopez C Peris-Martínez C. Subfoveal and parafoveal choroidal thickening in patients with keratoconus using the ETDRS grid on swept-source OCT. Ophthalmol Ther. (2024) 13:509–27. doi: 10.1007/s40123-023-00858-y, 38113025 PMC10787729

[14] Zeng Q Luo L Yao Y Tu S Yang Z Zhao M. Three-dimensional choroidal vascularity index in central serous chorioretinopathy using ultra-widefield swept-source optical coherence tomography angiography. Front Med (Lausanne). (2022) 9:967369. doi: 10.3389/fmed.2022.967369, 36160148 PMC9490028

[15] Zeng Y Liu M Li M Wei D Mao M Liu X . Early changes to retinal structure in patients with diabetic retinopathy as determined by ultrawide swept-source optical coherence tomography-angiography. Front Endocrinol (Lausanne). (2023) 14:1143535. doi: 10.3389/fendo.2023.1143535, 37223042 PMC10200911

[16] Curtin BJ Karlin DB. Axial length measurements and fundus changes of the myopic eye. I. The posterior fundus. Trans Am Ophthalmol Soc. (1970) 68:312–34. 5524211 PMC1310382

[17] Ohno-Matsui K Kawasaki R Jonas JB Cheung CMG Saw S-M Verhoeven VJM . International photographic classification and grading system for myopic maculopathy. Am J Ophthalmol. (2015) 159:877–883.e7. doi: 10.1016/j.ajo.2015.01.022, 25634530

[18] Zhou F Yuan Y Xu Y Huang Z Wang F Chen Z . Assessment of choriocapillary flow deficits in myopic children by optical coherence tomography angiography. Sci Rep. (2025) 15:15535. doi: 10.1038/s41598-025-98579-8, 40319126 PMC12049488

[19] Wang Q He L-B Li S Liu H-L Liu Y. Evaluating choroidal vascular changes in young adults with high myopia utilizing swept source optical coherence tomography angiography. Photodiagn Photodyn Ther. (2025) 51:104475. doi: 10.1016/j.pdpdt.2025.104475, 39788469

[20] Tang X Liang J Luo L Yuan F Zhao K Zhuo X . Investigation of choroidal vascular alterations in eyes with myopia using ultrawidefield optical coherence tomography angiography. BMJ Open Ophthalmol. (2024) 9:e001839. doi: 10.1136/bmjophth-2024-001839, 39567184 PMC11580270

[21] He X Sankaridurg P Naduvilath T Wang J Xiong S Weng R . Normative data and percentile curves for axial length and axial length/corneal curvature in Chinese children and adolescents aged 4-18 years. Br J Ophthalmol. (2023) 107:167–75. doi: 10.1136/bjophthalmol-2021-319431, 34531198 PMC9887397

[22] Benitez-Aguirre P Craig ME Cass HG Sugden CJ Jenkins AJ Wang JJ . Sex differences in retinal microvasculature through puberty in type 1 diabetes: are girls at greater risk of diabetic microvascular complications? Invest Ophthalmol Vis Sci. (2014) 56:571–7. doi: 10.1167/iovs.14-15147, 25477322

[23] Vujosevic S Limoli C Piccoli G Costanzo E Marenzi E Torti E . A gender-based analysis of retinal microvascular alterations in patients with diabetes mellitus using OCT angiography. J Diabetes Complicat. (2024) 38:108852. doi: 10.1016/j.jdiacomp.2024.108852, 39213716

[24] Zhang X Jiang J Kong K Li F Chen S Wang P . Optic neuropathy in high myopia: Glaucoma or high myopia or both? Prog Retin Eye Res. (2024) 99:101246. doi: 10.1016/j.preteyeres.2024.101246, 38262557

[25] Mu J Wei J Geng H Yi W Kang X Wen J . Wide-field swept-source optical coherence tomography angiography (SS-OCTA) in the assessment of retinal vessel density and thickness in 4-to 16-year-old myopic children. Photodiagn Photodyn Ther. (2024) 48:104240. doi: 10.1016/j.pdpdt.2024.104240, 38866069

[26] Geng H Mu J Wen J Yao W Liu L Liu C . Factors affecting choroidal circulation parameters in 4–14-year-old Chinese children measured by SS-OCT/OCTA. Photodiagn Photodyn Ther. (2024) 48:104275. doi: 10.1016/j.pdpdt.2024.104275, 39002833

[27] Jonas JB Jonas RA Bikbov MM Wang YX Panda-Jonas S. Myopia: histology, clinical features, and potential implications for the etiology of axial elongation. Prog Retin Eye Res. (2023) 96:101156. doi: 10.1016/j.preteyeres.2022.101156, 36585290

[28] Xu M Yu X Wan M Feng K Zhang J Shen M . Two-year longitudinal change in choroidal and retinal thickness in school-aged myopic children: exploratory analysis of clinical trials for myopia progression. Eye Vis (Lond). (2022) 9:5. doi: 10.1186/s40662-022-00276-4, 35101136 PMC8805434

[29] Wang X Chen Y Wang Z Li H He Q Rong H . Assessment of macular structures and vascular characteristics in highly myopic anisometropia using swept-source optical coherence tomography angiography. Front Physiol. (2022) 13:918393. doi: 10.3389/fphys.2022.918393, 36045745 PMC9421159

[30] Zhang Y Wang D Lin F Song Y Chen Y Peng Y . Diagnostic performance of wide-field optical coherence tomography angiography in detecting open-angle glaucoma in high myopia. Acta Ophthalmol. (2024) 102:e168–77. doi: 10.1111/aos.16603, 38129974

